# Antioxidant Supplementation in Childhood Obesity: A Path to Improved Metabolic Health?

**DOI:** 10.3390/antiox14040466

**Published:** 2025-04-13

**Authors:** Ancuta Lupu, Cristina Maria Mihai, Felicia Dragan, Irina Tarnita, Mirabela Alecsa, Tatiana Chisnoiu, Ionela Daniela Morariu, Magdalena Cuciureanu, Alin Horatiu Nedelcu, Delia Lidia Salaru, Emil Anton, Ciprian Danielescu, Silvia Fotea, Gabriela Stoleriu, Omer Faruk Beser, Vasile Valeriu Lupu

**Affiliations:** 1 Pediatrics, “Grigore T. Popa” University of Medicine and Pharmacy, 700115 Iasi, Romania; ancuta.ignat1@umfiasi.ro (A.L.); mirabela-smaranda.alecsa@umfiasi.ro (M.A.); vasile.lupu@umfiasi.ro (V.V.L.); 2 Pediatrics, Faculty of Medicine, “Ovidius” University, 900470 Constanta, Romania; cristina_mihai@365.univ-ovidius.ro (C.M.M.); tatiana.chisnoiu@365.univ-ovidius.ro (T.C.); 3 Faculty of Medicine and Pharmacy, University of Oradea, 410087 Oradea, Romania; 4 Faculty of Pharmacy, “Grigore T. Popa” University of Medicine and Pharmacy, 700115 Iasi, Romania; ionela.morariu@umfiasi.ro; 5 Faculty of Medicine, “Grigore T. Popa” University of Medicine and Pharmacy, 700115 Iasi, Romania; mag.cuciureanu@umfiasi.ro (M.C.); alin.nedelcu@umfiasi.ro (A.H.N.); delia.salaru@umfiasi.ro (D.L.S.); emil.anton@umfiasi.ro (E.A.); ciprian.danielescu@umfiasi.ro (C.D.); 6 Clinical Medical Department, Faculty of Medicine and Pharmacy, “Dunarea de Jos” University of Galati, 800008 Galati, Romania; silvia.fotea@ugal.ro (S.F.); gabriela.stoleriu@ugal.ro (G.S.); 7 Department of Pediatric Gastroenterology, Hepatology & Nutrition, Cerrahpasa Medical Faculty, Istanbul University Cerrahpasa, 34776 Istanbul, Turkey; ofbeser@gmail.com

**Keywords:** antioxidants, obesity, children, oxidative stress, supplementation

## Abstract

Childhood obesity is linked to heightened oxidative stress, a key driver of endothelial dysfunction, inflammation, and metabolic complications. Antioxidants, including Vitamins C and E, are vital in neutralizing free radicals and mitigating oxidative damage. This non-systematic review examines the potential advantages of antioxidant supplementation in pediatric obesity, focusing on its effects on vascular health, insulin sensitivity, and inflammatory processes. Emerging data suggest that antioxidants may improve endothelial function, reduce blood pressure, and enhance metabolic homeostasis in obese children. However, the long-term efficacy and safety of antioxidant supplementation remain uncertain, necessitating further rigorous randomized controlled trials. A deeper understanding of antioxidants’ role in pediatric obesity could unlock novel therapeutic approaches for managing obesity-related complications and improving children’s overall health outcomes.

## 1. Introduction

Childhood obesity is a significant global health issue, with its prevalence nearly tripling since 1975; the World Health Organization (WHO) reported over 39 million children under five classified as overweight or obese in 2020 [1]. A child is considered overweight with a body mass index (BMI) at or above the 85th percentile and obese at or above the 95th percentile for their age and sex. This alarming trend is driven by a complex interplay of genetic, environmental, and behavioral factors, such as increased consumption of energy-dense foods and sedentary lifestyles [2,3]. Beyond immediate health implications, childhood obesity is a strong predictor of adult obesity and chronic diseases like cardiovascular disease and type 2 diabetes [4].

A critical yet often underexplored consequence of childhood obesity is its relationship with oxidative stress (OS) and metabolic dysfunction, where OS contributes to complications like insulin resistance, dyslipidemia, and systemic inflammation [5,6]. Excess adipose tissue induces low-grade inflammation, increasing reactive oxygen species (ROS) production through processes like mitochondrial dysfunction and altered lipid metabolism [7]. This can impair endothelial function and contribute to atherosclerosis [8]. Research indicates that OS markers, including lipid peroxidation and protein carbonylation, are significantly elevated in obese children compared to their non-obese peers [9,10].

Various intervention strategies have been explored, including dietary changes, increased physical activity, and behavioral therapy. Recently, researchers have focused on antioxidants, which may play a complementary role in managing childhood obesity by mitigating oxidative damage and inflammation [11,12]. Dietary antioxidants, such as vitamins C and E, are suggested to improve metabolic health, though their long-term efficacy compared to established interventions remains under investigation [13,14]. Some argue that sustained lifestyle changes yield better long-term health outcomes than antioxidant supplementation alone [15,16], particularly as puberty introduces natural insulin resistance that can affect OS and inflammation [17,18].

Pediatric obesity triggers chronic low-grade inflammation through various molecular pathways, including increased cytokine production and immune cell infiltration into adipose tissue, predominantly involving pro-inflammatory M1 macrophages [19,20,21,22,23,24]. This pro-inflammatory environment leads to amplified OS and further increases ROS production, exacerbating inflammation and contributing to metabolic syndrome [25,26,27,28,29,30].

Ultimately, pediatric obesity and metabolic syndrome are associated with a heightened pro-inflammatory state and disrupted redox homeostasis [31,32,33,34]. This review critically examines the relationship between childhood obesity and OS, evaluating the potential of antioxidant therapy as a targeted intervention. It will address key epidemiological trends while assessing the limitations and inconsistencies in current research on antioxidants in childhood obesity. By comparing antioxidant therapy to conventional approaches such as dietary modifications and physical activity, this review aims to clarify the role of OS in childhood obesity and determine whether antioxidant therapy can serve as a viable complement to traditional treatment methods. The findings may guide future research directions and inform public health policies. The current literature on antioxidant supplementation in childhood obesity reveals methodological limitations, including small samples and inconsistent findings, underscoring the need for large-scale, randomized controlled trials to enhance the reliability of results.

Recognizing the global impact of obesity and its comorbidities in both children and adults, this review will deepen the understanding of the mechanisms involved and explore strategies for addressing these challenges. Antioxidants can be categorized into endogenous and exogenous types, with both being essential; however, addressing nutritional deficiencies in overweight individuals is critical for restoring adequate levels and ensuring the effective functioning of endogenous antioxidants. We plan to conduct a narrative review of the scientific literature from recent decades by accessing international databases. To streamline our search process, we will use keywords such as “obesity”, “oxidative stress”, “antioxidant enzymes”, “exogenous antioxidants”, “antioxidant vitamins”, “antioxidant trace minerals”, and “herbal medicines used as antioxidants”.

## 2. Exogenous Antioxidants and Their Implications in Pediatric Obesity

**Figure 1:**
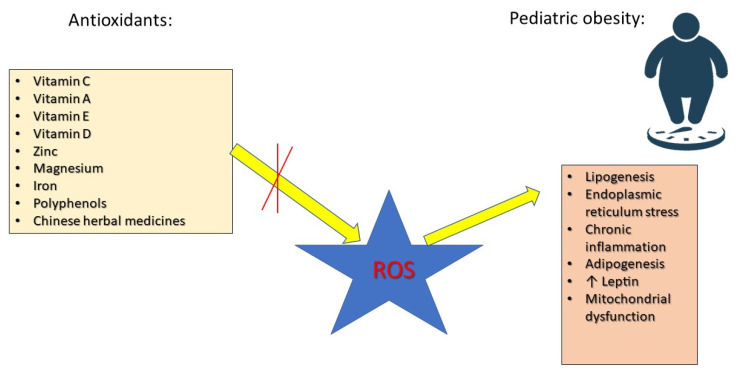
The roles of antioxidants in preventing the effects of ROS in pediatric obesity.

Antioxidants play a crucial role in neutralizing ROS, preventing cellular damage, and improving metabolic function. Research has focused on various antioxidants, including vitamin C, which improves endothelial function, reduces inflammation, and enhances insulin sensitivity [16]. Vitamin E prevents lipid peroxidation, reduces NF-κB activation, and supports cardiovascular health [35]. Vitamin A regulates adipocyte differentiation and lipid metabolism via peroxisome proliferator-activated receptor (PPAR) activation [36]. Selenium and zinc are essential for the activity of glutathione peroxidase (GPx) and superoxide dismutase (SOD), key enzymes in OS defense [37]. Coenzyme Q10 (CoQ10) and polyphenols improve mitochondrial efficiency and energy metabolism [38]. Research has demonstrated a connection between overweight and obesity and elevated levels of inflammatory markers, such as tumor necrosis factor alpha (TNF-α), interleukins (ILs) 6 and 4, leptin, and macrophage chemoattractant protein-1. Additionally, ROS play a crucial role in the onset of obesity and its associated complications. As a result, antioxidant agents like carotenoids, vitamin E, vitamin C, zinc, magnesium, and selenium may offer protective benefits against the negative consequences of obesity and being overweight [39] (Figure 1).

### 2.1. Vitamins as Antioxidants

#### 2.1.1. Vitamin C

Vitamin C (C_6_H_8_O_6_) is a crucial nutrient necessary for the proper operation of numerous metabolic functions within the body [40]. It plays a vital role in the production of collagen, carnitine, and several neurotransmitters, and serves as a significant water-soluble antioxidant that effectively neutralizes free radicals. Additionally, as a reducing agent, vitamin C enhances the absorption, transportation, and retention of iron. Moreover, it is essential for optimal function of the immune system. At a physiological pH, ascorbic acid (AscH2) dissociates into its resonance-stabilized anion known as ascorbate (AscH−) [41]. Ascorbate serves as a potent reducing agent capable of donating electrons to various molecules, functioning as both a non-enzymatic antioxidant and a co-factor for enzymes that depend on transition metals. It helps maintain redox potential by transferring electrons or protons between its ascorbyl radical form (semidehydroascorbate, SDA) and dehydroascorbic acid [41].

Research involving animals has shown that the intake of vitamin C can influence insulin sensitivity in hyperglycemic ob/ob mice without impacting their body weight [42]. Additionally, vitamin C supplementation has been linked to a notable decrease in fat deposits [43]. Positive outcomes from vitamin C administration have also been reported in models of induced obesity [44,45].

Extensive research suggests that diets rich in fruits, vegetables, and whole foods can significantly affect body composition and overall health. Generally, it has been acknowledged that diets incorporating a greater proportion of fruits and vegetables tend to be linked with a lower risk of weight gain. Furthermore, it has been emphasized that vitamin C plays a crucial part in regulating body weight and fat [46].

Body mass is a key factor in determining vitamin requirements, including vitamin C. Recommendations for vitamin C intake are typically based on data from healthy-weight males and then adjusted for other groups by age and sex. Additionally, it is plausible that weight gain associated with obesity could influence vitamin C requirements. A comprehensive review of existing literature was conducted to evaluate the evidence regarding the impact of obesity on vitamin C consumption or levels [47]. This section of the review highlights the complex relationship between obesity and vitamin C status, suggesting that obesity may increase the body’s requirement for this vitamin. Numerous observational studies have shown lower vitamin C levels in overweight and obese individuals, potentially due to reduced dietary intake. However, intervention studies reveal that even with identical vitamin C supplementation, obese individuals maintain lower serum levels compared to those of normal weight. This finding suggests that obesity itself impacts vitamin C metabolism, beyond simple dietary differences.

Several hypotheses attempt to explain this phenomenon: altered vitamin C distribution between lean and fat tissues, dilution effects due to increased body volume, obesity-related metabolic changes, and dysbiosis of the gut microbiota. Further research is needed to clarify these mechanisms. Specifically, the authors recommend depletion–repletion studies and pharmacokinetic analyses across a wide range of body weights and ages to determine whether obesity truly increases the requirement for vitamin C [47].

Furthermore, emerging research reveals a novel role for vitamin C in gene expression and epigenetics. Vitamin C regulates hypoxia-inducible factor hydroxylases [48] and exerts epigenetic effects by modulating Ten-Eleven Translocation DNA demethylases and histone demethylases [49]. These findings warrant further investigation into vitamin C’s role in the context of obesity-related gene expression and epigenetic modifications. The significance of vitamin C is exemplified by its deficiency, which can lead to scurvy—a serious condition marked by dysfunction in connective tissues, mood disturbances, weakened immune response, and muscle weakness that can ultimately prove fatal. Even less severe deficiencies can cause fatigue and a persistent sense of tiredness, adversely impacting quality of life and work performance [40].

In numerous countries, vitamin C intake guidelines have been formulated based on limited pharmacokinetic studies of healthy young individuals with a normal BMI [50,51]. However, recent findings indicate that individuals with higher body weights may exhibit lower plasma concentrations of vitamin C. Various mechanisms could contribute to this observation. For instance, obese individuals might have lower vitamin C intake due to distinct dietary habits, leading to reduced vitamin C status. Additionally, the increase in overall body size associated with obesity can result in volumetric dilution of vitamin C, though this effect may be partially balanced by how nutrients are distributed between lean and fat tissues. Furthermore, the basal metabolic rate tends to rise in obese individuals due to increased body mass, which affects the demand for vitamin C. Lastly, metabolic changes linked to obesity may influence vitamin C status, impacting its metabolism, absorption, and excretion, while imbalances in gut microbiota may also disrupt the absorption or metabolism of this important vitamin.

Research involving children has revealed an interesting connection between higher vitamin C intakes and obesity. An analysis of NHANES III data regarding boys and girls aged 12 to 16 showed that overweight boys were more likely to meet their vitamin C intake requirements compared to their normal weight or underweight peers; however, no significant difference in vitamin C intake based on weight status was observed among girls [52]. In another study conducted in Brazil, children who were overweight or diagnosed with obesity had vitamin C intakes that were twice as high as those of children with a healthy weight [53]. This situation may suggest that the availability and consumption of vitamin C-rich fruit juices contributed to the rising rates of obesity among children, even as it improved their vitamin C intake. Conversely, a cross-sectional study carried out in Tehran, Iran, involving 356 children found that a diet rich in vitamin C and phytochemicals was linked to a lower risk of obesity in both crude and adjusted models [54].

Two studies conducted in rural Mexico examined the links between obesity and vitamin C status in healthy females and school-aged children. In the study involving females aged 25 to 55, researchers found no significant difference in plasma AscH2 concentrations across various BMI categories. However, linear regression analysis revealed inverse relationships between both BMI and waist-to-height ratio with vitamin C status; intriguingly, factors such as body fat, waist circumference, and abdominal fat did not show associations with vitamin C status [55]. Similarly, in the cohort of Mexican children, an inverse relationship was noted between vitamin C status and waist-to-height ratio, body fat, and abdominal fat [56]. While other obesity measures in these two groups demonstrated a similar trend of inverse association with vitamin C status, these findings did not reach statistical significance [55,56].

In a double-blind, randomized study [56], children affected by obesity aged 8 to 12 were divided into two groups: one received 500 mg of vitamin C and the other a placebo for 45 days, with eight lean controls also assessed. The study found that children diagnosed with obesity had higher mean blood pressure (MBP) and lower forearm vascular conductance (FVC) compared to lean controls. After 45 days of vitamin C supplementation, MBP significantly decreased, and FVC improved both at rest and during mental stress. By the end of the intervention, vascular function in the vitamin C group was similar to that of lean controls. These findings suggest that vitamin C may help improve vascular health in children with obesity by enhancing blood flow regulation and reducing blood pressure. The study by Fernandes et al. [57] demonstrates that vitamin C not only restores blood pressure levels but also enhances the vasodilatory response during mental stress in obese children, suggesting its potential therapeutic role in managing cardiovascular risk factors in this vulnerable population [57].

It is established that an optimal intake of vitamin C for an individual weighing around 60 kg is 110 mg per day. Carr AC et al. [58] highlight that to prevent vitamin C deficiencies, supplementation should include an additional 10 mg for every 10 kg of excess body weight [58,59].

#### 2.1.2. Vitamin A

Vitamin A (C_20_H_30_O), an essential fat-soluble nutrient, plays a vital role in numerous facets of human health and growth. It is a key dietary micronutrient that participates in several physiological functions, such as immune system support, development, and metabolic processes [60,61]. Research indicates that vitamin A significantly impacts fat metabolism, which is important for managing body weight [62,63,64].

However, factors such as dietary intake and lifestyle habits can influence serum concentrations of this essential nutrient. Research indicates that individuals with obesity often face vitamin A deficiency (VAD) [65,66]. The cause of this deficiency remains unclear, as it could result from inadequate dietary intake or other factors related to obesity, such as OS and inflammation [67]. Studies show a negative correlation between BMI and serum levels of β-carotene, β-cryptoxanthin, retinol, and other carotenoids (α-carotene, lutein + zeaxanthin) [67,68]. Even with similar carotenoid intake across BMI groups, obese individuals exhibit lower serum levels of these vitamin A precursors. Conversely, higher serum levels of α-carotene, trans-β-carotene, and cis-β-carotene are associated with a lower risk of overweight and obesity in children, while elevated retinol levels are linked to a higher risk [69,70]. This inverse relationship between serum carotenoids and obesity might be explained by differences in fruit and vegetable consumption and overall energy intake between obese and non-obese individuals [71]. While obese individuals may consume excessive energy-rich foods, they might still fail to meet their micronutrient requirements. Additionally, compared to those with normal weight, obese individuals may store more β-carotene in their adipose tissue, leading to lower serum carotenoid concentrations [72,73].

A relevant investigation by Libien et al. [74] highlighted how vitamin A and its derivatives are involved in adipose tissue metabolism [74]. Insufficient levels of vitamin A have been associated with a higher risk of obesity, affecting body weight management via its metabolic mechanisms [67,70]. Additionally, there seems to be a link between vitamin A serum levels and BMI, with those who were diagnosed with obesity often showing reduced serum concentrations of vitamin A compared to individuals of normal weight [75]. Furthermore, studies conducted both in vitro and in vivo have shed light on the functions of vitamin A derivatives, including retinal and retinoic acid, as well as their regulatory proteins related to adipose tissue metabolism, which are significant in understanding the development of obesity.

The effects of weight loss on fat-soluble vitamin status in children with obesity were investigated by Joanna Gajewska et al., focusing on the relationship between vitamin concentrations and factors such as dietary intake, body measurements, and adipokines [76]. Conducted in 60 children affected by obesity, the research measured vitamin A and E levels, utilizing high-pressure liquid chromatography before and after a weight loss intervention. The study measured retinol-binding protein 4 (RBP4), leptin, soluble leptin receptor, and high-molecular-weight adiponectin levels using immunoenzymatic assays. Post-therapy, vitamin E intake decreased, while vitamin A/lipid and vitamin E/lipid ratios increased, correlating with reduced triglyceride levels. Changes in vitamin levels showed a positive correlation with both dietary intake and adipokine ratios. RBP4 levels, however, correlated with BMI and cholesterol changes. The study concludes that weight loss in children may elevate the risk of vitamin E deficiency and underscores the impact of BMI on vitamin A status [76].

A common health issue among children in developing nations is VAD [77]. Research by Lin LM et al. [78] indicated that VAD is prevalent among Chinese children, particularly in isolated and impoverished rural regions where dietary interventions or, in some cases, vitamin A supplementation are necessary [78]. Studies have reported low serum retinol levels in individuals who are overweight or obese [79]. Furthermore, a case-control investigation found a negative relationship between serum retinol levels and weight, BMI, and hip circumference among adults with overweight or obesity in Thailand [80]. VAD in the human population is primarily driven by inadequate dietary intake and, in some cases, excessive consumption associated with factors like acute infections. Research has shown significant associations between dietary vitamin A levels and plasma retinol concentrations, particularly among preschool children in Chongqing, where unbalanced diets lead to nutrient insufficiency. In school-aged children, lower vitamin A levels were linked to obesity, with children affected by obesity exhibiting a 2.37-fold higher risk of VAD compared to their normal-weight counterparts. This relationship suggests that poor dietary habits may hinder vitamin A intake or absorption, which in turn contributes to the development of obesity. Furthermore, vitamin A is noted to play a vital role in regulating inflammatory responses and may influence body weight through its metabolites [78].

#### 2.1.3. Vitamin E

Vitamin E (C_29_H_50_O_2_), a fat-soluble dietary antioxidant, is essential for energy metabolism, influencing the expression of various genes. Among the eight isomers of vitamin E, the most commonly consumed forms are α-tocopherol and γ-tocopherol [81]. While γ-tocopherol is primarily recognized for its antioxidant capabilities, α-tocopherol plays a significant role in the regulation of genes related to diverse cellular functions. Vitamin E exhibits both anti-inflammatory and antioxidant effects. Inflammatory cytokines such as TNF-α, IL-1β, and IL-6 can reduce insulin sensitivity and promote obesity. Vitamin E effectively mitigates the levels of pro-inflammatory cytokines and removes free radicals due to its antioxidant properties. Research indicates that α-tocopherol promotes the expression of adiponectin by activating PPAR-γ, the key regulator of adiponectin production. Adiponectin, a hormone produced by adipose tissue, plays a significant role in managing body composition and energy metabolism by influencing appetite [81]. This adipokine enhances fatty acid oxidation in muscle cells, inhibits glucose production in the liver, and improves insulin sensitivity. Therefore, vitamin E may help regulate energy balance and body weight by increasing adiponectin levels. Furthermore, there is substantial evidence that vitamin E helps to lower cholesterol levels. Tocotrienols inhibit 3-hydroxy-3-methylglutaryl-coenzyme A reductase (HMG-CoA reductase), an enzyme in the liver responsible for cholesterol synthesis. Consequently, vitamin E can prevent the buildup of cholesterol, which may lead to obesity by exacerbating inflammatory responses and increasing IR [80]. Overall, vitamin E has the potential to mitigate obesity through various biological pathways. However, despite these proposed mechanisms, clinical trials have produced mixed results concerning the effects of vitamin E supplementation on body weight.

Chronic low-grade inflammation in individuals affected by obesity is associated with lower levels of adiponectin. Adiponectin, also recognized as ACRP30 or AdipoQ, is a cytokine secreted primarily by adipose tissue [82]. Elevated adiponectin levels promote glucose uptake and suppress gluconeogenesis [83,84], and also increase fatty acid oxidation while reducing lipid peroxidation caused by OS [85]. Vitamin E, a potent lipid-soluble antioxidant, prevents lipid peroxidation in cell membranes. One study showed that 400 IU of daily vitamin E supplementation improved endothelial function in children [86]. Vitamin E might increase adiponectin levels through several mechanisms: its anti-inflammatory properties may counteract the low-grade inflammation in obesity by suppressing pro-inflammatory cytokines, thereby potentially increasing adiponectin production. Furthermore, vitamin E’s structural similarity to PPAR-γ ligands suggests that it may activate the PPAR-γ-responsive promoter region of the adiponectin gene, leading to increased adiponectin expression [87,88,89]. However, data on the benefits of vitamin E supplementation in obese children are limited. This study investigates the effects of vitamin E supplementation on lipid profiles and adiponectin levels in obese adolescents.

A randomized, double-blind, controlled trial was conducted involving adolescents with obesity aged 14 to 18 years who had no prior use of anti-obesity or antioxidant medications. Participants were randomly assigned to one of two groups: one receiving vitamin E and the other a placebo. The vitamin E dosage was set at 400 IU per day, and the intervention lasted for two months. Lipid profiles and adiponectin levels were assessed at both baseline and following the intervention. Primary outcomes were evaluated based on the per-protocol analysis approach. Statistical comparisons were made using independent *t*-tests or the Mann–Whitney U test. In total, 66 participants completed the study—34 in the vitamin E group and 32 in the placebo group. The results revealed no significant differences in both lipid profiles and adiponectin levels between the two groups at the two-month mark after the intervention [90].

Another study explored the connections between antioxidant and micronutrient levels and body fat in Mexican-American children, using data from the NHANES 2001–2004 surveys, where micronutrient deficiencies were found to be low. It was observed that higher serum concentrations of certain carotenoids (α-carotene, trans-β-carotene, cis-β-carotene) and cholesterol-adjusted α-tocopherol were associated with a reduced risk of childhood overweight or obesity, whereas higher retinol concentrations correlated with an increased risk. Additionally, these carotenoids and α-tocopherol were inversely related to overall fat mass and trunk fat mass. Thus, the study supports the hypothesis that the status of antioxidant micronutrients is linked to body adiposity in this population [72].

#### 2.1.4. Vitamin D

Vitamin D deficiency, defined by 25-hydroxyvitamin D [25(OH)D] levels below 20 ng/mL, is associated with several chronic conditions, including IR, MetS, atherosclerosis, and obesity [91,92]. Vitamin D is primarily produced endogenously in the skin through sun exposure, with limited dietary sources (Table 1) [93,94]. Low vitamin D status in obese individuals may result from insufficient dietary intake, reduced sun exposure due to decreased physical activity, and impaired absorption of dietary vitamin D, particularly following bariatric surgery [95,96]. Geographic location also plays a role; populations in high latitudes with limited sun exposure exhibit higher obesity rates and lower endogenous vitamin D production [97].

The increasing prevalence of obesity in children and adolescents is linked to vitamin D deficiency [98]. Children with inadequate vitamin D intake (<70 IU/day) tend to have higher body weight, BMI, waist circumference, and waist-to-hip ratio [99]. This association is also observed in adolescents, although parathyroid hormone levels do not consistently increase [100]. The relationship between vitamin D deficiency and obesity in this age group may involve metabolic and hormonal factors [101,102]. Excess body fat can influence vitamin D metabolism in several ways: it may enhance the clearance of vitamin D, decrease its bioavailability in adipose tissue, or dilute its concentration due to increased body mass [103,104]. Lean children typically have total body fat masses that vary by age, while children with overweight or obesity have more than three times that amount [105]. Adipose tissue is now recognized as an active endocrine organ that secretes many proteins and plays a role in glycemic control [106,107,108]. Although vitamin D interacts with adipose tissue and affects metabolic processes, the specific molecular mechanisms remain unclear.

Both active vitamin D (1,25(OH)2D) and vitamin D3 can influence adipogenic genes and transcription factors, but their exact roles in fat accumulation are complex and sometimes contradictory [106,109,110]. Additionally, vitamin D metabolites can modulate cytokine production and the inflammatory response in fat tissue, which is linked to obesity-related metabolic issues like IR and liver fat deposition. As fat cells expand, they release pro-inflammatory cytokines such as TNF-α and IL-6 [106]. Observational studies indicate a negative correlation between 25(OH)D levels and inflammatory cytokines, with evidence suggesting that 25(OH)D may help reduce their production [111,112,113,114,115].

Children and adolescents affected by obesity are at heightened risk for vitamin D deficiency due to factors such as increased adipose tissue dilution and insufficient dietary intake [116,117,118,119]. Low vitamin D levels can worsen metabolic profiles and are linked to conditions like IR and a greater likelihood of developing MetS [120,121,122]. Despite interest in vitamin D supplementation, the effectiveness in restoring levels and reducing cardiovascular risk factors remains unclear [123,124,125], leading to a lack of clear recommendations for usage in this population.

A systematic review from 2017 assessed the effects of vitamin D supplementation in non-obese and children affected by obesity, finding no significant improvement in vitamin D levels for those receiving supplements compared to placebo, although supplementation was more effective in non-obese children [126]. While some studies showed increases in vitamin D levels among children with overweight and obesity after supplementation, the clinical relevance of these increases was questioned, with low percentages of children affected by obesity achieving vitamin D sufficiency. For example, only 50% of children diagnosed with obesity normalized their vitamin D levels compared to 89% of non-obese children [127]. The Italian Pediatric Society recommends higher doses of vitamin D for children affected by obesity (1000–1500 IU daily) during fall and winter or year-round in cases of inadequate sun exposure [128,129].

Due to the high rates of vitamin D deficiency among children diagnosed with obesity, their supplementation needs are likely different from standard recommendations. The AAP suggests 5000 IU daily for children with deficiencies, and our research supports this higher dose, showing significant improvement in vitamin D levels among adolescents with obesity [130,131]. Current recommendations from the Endocrine Society and various studies indicate that higher doses of vitamin D may be necessary for individuals with obesity [132,133]. Children diagnosed with obesity exhibit reduced efficiency in utilizing vitamin D, which means they require more supplementation to achieve similar increases in serum vitamin D levels compared to their lean counterparts [131,134,135]. These findings highlight the need for tailored guidelines regarding vitamin D supplementation for youths with obesity [136].

Although direct evidence linking vitamin D to obesity and metabolic parameters is limited, inflammation may mediate the observed associations [137]. Vitamin D may suppress inflammatory responses, and given that obesity is a state of chronic, low-grade systemic inflammation, this interaction is relevant [138,139]. Some studies suggest that raising 25(OH)D levels might improve weight loss outcomes, possibly due to vitamin D’s anti-inflammatory effects. However, a meta-analysis of randomized controlled trials in adults found no effect of vitamin D supplementation on inflammatory biomarkers in overweight or obese individuals, highlighting the need for further research. Recent studies suggest that vitamin D supplementation might reduce inflammatory biomarkers, such as IL-6, in adults with MetS, but more randomized controlled trials in obese children and adolescents are needed [139,140,141]. Obesity-related vitamin D deficiency might also stem from reduced bioavailability of dietary vitamin D due to its sequestration in adipose tissue. Some liquid chromatography-mass spectrometry (LC-MS/MS) studies have detected vitamin D3 concentrations in subcutaneous adipose tissue of obese individuals that are more than ten times higher than serum levels [142,143,144]. This accumulation of the fat-soluble vitamin D in adipose tissue may limit its availability for hepatic conversion to 25(OH)D, resulting in lower plasma 25(OH)D levels in individuals with significant adiposity.

### 2.2. Microelements

#### 2.2.1. Magnesium

Magnesium (Mg) plays a crucial role as a cofactor for enzymes responsible for processing carbohydrates. Research has shown a significant connection between Mg levels and insulin function [145,146]. In adults, insufficient Mg levels in the blood and cells have been linked to IR, poor glucose regulation, and reduced insulin production [147,148,149]. Additionally, extensive population studies suggest that inadequate Mg intake and lower blood Mg levels are associated with a higher likelihood of developing type 2 diabetes [150,151]. Mg deficiency is commonly associated with key metabolic risk factors, including hyperlipidemia, hypertension, diabetes, and obesity. As a crucial divalent metal ion, Mg functions as a cofactor for enzymes involved in fat, protein, and carbohydrate metabolism, while also playing a key role in insulin function. It is essential for enzymes like lecithin cholesterol acyltransferase and lipoprotein lipase, which regulate triglyceride levels and HDL-cholesterol (HDL-C), and it influences cholesterol biosynthesis through Mg (2+)-ATP. Additionally, Mg impacts glucose and insulin metabolism by modulating tyrosine kinase activity, facilitating glucose transport into cells, and influencing glycogen breakdown. Hypomagnesemia has also been linked to OS, though it remains unclear whether it is a cause or consequence of metabolic dysfunction. While studies have consistently associated obesity with lower serum Mg levels and suggested Mg deficiency may contribute to metabolic disorders, the precise relationship between Mg and obesity remains uncertain. Serum Mg is considered a reliable indicator of Mg status, correlating well with intracellular Mg levels measured via nuclear magnetic resonance spectroscopy [152].

Mg is essential for numerous biochemical processes, including glucose metabolism, where it acts as a cofactor for enzymes involved in oxidative pathways. In obese individuals, low intracellular Mg levels can impair glucose oxidation and shift metabolism towards the pentose phosphate pathway, resulting in excessive NADPH production that fosters triglyceride synthesis, adipocyte fat storage, and exacerbates obesity. This, in turn, increases the risk of dyslipidemia and IR. Moreover, Mg is pivotal in the synthesis and activation of vitamin D, which plays a role in immune modulation and the regulation of the renin-angiotensin system. Deficient Mg status can impair vitamin D activation, further contributing to cardiometabolic risks. Several studies suggest that optimizing Mg levels may enhance vitamin D’s beneficial effects on metabolic health, reducing the likelihood of obesity-related comorbidities. Therefore, the interplay between Mg and vitamin D in obesity underscores their combined influence on the pathogenesis and management of metabolic diseases [153].

However, the impact of Mg deficiency on IR in children still needs more investigations. One study revealed a significant difference in both serum Mg levels and dietary Mg intake between lean children and those affected by obesity, indicating a potential role of Mg deficiency in metabolic dysfunction. Children diagnosed with obesity exhibited significantly lower serum Mg concentrations and consumed less dietary Mg compared to their lean counterparts. Furthermore, an inverse relationship was observed between serum Mg levels and fasting insulin, suggesting that lower Mg levels may contribute to hyperinsulinemia. Additionally, a positive correlation between Mg levels and the quantitative insulin sensitivity check index highlights the potential influence of Mg status on insulin sensitivity. These findings suggest that Mg deficiency could be a contributing factor in the early development of IR in pediatric obesity, warranting further investigation into its underlying mechanisms and potential therapeutic interventions [154].

Another case-control study [155] examined the relationship between serum Mg levels and body weight in children aged 2–14 years. Conducted over 12 months, it included 140 children divided into overweight/obese (cases) and normal-weight (controls) groups. Results showed significantly lower serum Mg levels in children categorized as overweight and obesity compared to those who were normal weight, with a strong inverse correlation between Mg levels and BMI, suggesting a potential link between Mg deficiency and higher body weight [155]. Moreover, a study compared 50 children with obesity with 50 normal-weight controls and found that children diagnosed with obesity had lower serum Mg and HDL-C levels, as well as higher total cholesterol, LDL-C, triglycerides, and blood pressure. A strong inverse correlation was observed between serum Mg and the degree of obesity, while serum Mg also showed a moderate inverse correlation with total cholesterol and LDL-C. The degree of obesity was positively correlated with total cholesterol and LDL-C but not with triglycerides or HDL-C [152].

#### 2.2.2. Zinc

Zinc is a vital trace element and an integral part of numerous enzymes, playing a key role in the synthesis, storage, and release of insulin. Both experimental and clinical studies have highlighted that zinc deficiency may contribute to the development of glucose intolerance, diabetes mellitus, IR, atherosclerosis, and coronary artery disease. Additionally, the impact of zinc on lipid profiles, particularly LDL-C and HDL-C, has been documented, suggesting its role in modulating lipid metabolism [156,157].

One randomized, placebo-controlled crossover trial, conducted in 2008 with 60 Iranian children with obesity, aimed to assess the impact of zinc supplementation on cardiometabolic risk factors [156]. Participants were randomly divided into two groups: one received 20 mg of elemental zinc daily for eight weeks, while the other group received a placebo. After a 4-week wash-out period, the groups crossed over. The study found that zinc supplementation significantly improved fasting plasma glucose, insulin levels, and insulin sensitivity (HOMA-IR) in children affected by obesity, but had no effect on BMI, waist circumference, LDL-C, or triglycerides. It suggests that zinc, alongside lifestyle changes, could be a safe and effective adjunctive treatment for reducing cardiometabolic risk factors related to childhood obesity [156].

In a study conducted from March to April 2018, primary data were collected on anthropometric measurements, nutritional intake, and blood sugar levels, along with serum lipid profiles (total cholesterol, triglycerides, HDL-C, and LDL-C), before and after zinc supplementation. The zinc supplement, administered as a 20 mg dose (1 teaspoon) of syrup daily, was provided to children affected by obesity and overweight aged 6–12 years from the Bugis-Makassar ethnic group, all of whom were physically healthy and had parental consent. Zinc supplementation significantly reduced blood sugar levels, total cholesterol, and HDL-C. However, while triglyceride and LDL-C levels decreased, the changes were not statistically significant [158].

A disruption in zinc metabolism has been observed in individuals with obesity; Marreiro, D.D.N. et al. [159] aimed to explore whether zinc nutritional status is linked to hyperinsulinemia in obesity. The study included 23 children and adolescents diagnosed with obesity (aged 7–14 years), compared to a control group of 21 age-matched individuals. Dietary information was collected using 3-day food records, and body composition was assessed through BMI, bioelectrical impedance, and skinfold measurements. Zinc status was evaluated by measuring zinc concentrations in plasma, erythrocytes, and 24 h urine samples. Both groups had marginal zinc concentrations in their diets. Zinc levels in plasma and erythrocytes were significantly lower in the obese group. Additionally, urinary zinc excretion and serum insulin levels were significantly higher in the obese group. However, there was no significant correlation between insulin levels and zinc nutritional status. Given that zinc plays a crucial role in the synthesis and secretion of insulin, the findings suggest a need for further investigation into the potential involvement of zinc in IR, a common feature of obesity [159].

Maintaining zinc levels within the normal range is crucial, as even a slight deficiency can increase the risk of obesity [140]. Mendes Garrido Abregú F. et al. [160] highlight the importance of adequate zinc intake during breastfeeding, noting that proper zinc nutrition after weaning can help prevent cardio-metabolic issues linked to perinatal restriction [160,161].

#### 2.2.3. Selenium

Selenium is a vital element and a cofactor for several enzymes with antioxidant functions, including GPx. A deficiency in selenium can disrupt cellular antioxidant capacity and contribute to the development of various health issues. Selenium supplementation has been shown to positively affect body weight regulation, metabolic functions, and OS, much like zinc [162,163]. In addition to acting as a vital cofactor for the antioxidant enzyme GPx, selenium also has anti-inflammatory properties and helps protect against non-alcoholic fatty liver disease caused by obesity. It achieves this by promoting the production of selenoprotein P1, which regulates the KEAP1/Nrf2 pathway [164,165]. The benefits of selenium exposure have also been confirmed by Abo El-Magd NF. et al. [166]. Furthermore, manganese plays an essential role in maintaining the body’s antioxidant defense system by serving as a redox-active component crucial for responding to OS. It primarily functions as a cofactor for SOD and a substrate for manganese-based non-protein antioxidants, with iron acting as the opposing element in these processes [167,168,169,170,171,172].

Selenium, as a component of essential selenoproteins like GPx and TrxR, plays a pivotal role in neutralizing free radicals and defending cells against OS. This function is crucial for maintaining cellular redox balance, regulating cell processes like signal transduction, proliferation, and aging, and protecting against diseases such as cancer, cardiovascular conditions, and neurodegenerative disorders. Selenium’s advantage over sulfur in selenocysteine, with faster kinetics and better reversibility in oxidation–reduction reactions, strengthens its role in cellular antioxidant defense. Beyond antioxidant defense, selenium also contributes to DNA repair, thyroid hormone metabolism, immune function, and reproductive health. Additionally, selenium nanoparticles, with their enhanced bioavailability, reduced toxicity, and superior therapeutic potential, show promise in biomedicine for managing OS, inflammation, and preventing diseases like cancer and cardiovascular diseases. This underscores selenium’s critical balance in the body, emphasizing the harm caused by both deficiency and excess, highlighting its importance for health and disease prevention.

Individuals categorized as overweight or obese often exhibit diminished antioxidant protection and increased oxidative stress compared to their counterparts with normal body weight. This vulnerability is further compounded when combined with other risk factors, particularly due to insufficient intake of antioxidant nutrients such as selenium, which is especially critical for children whose rapid growth elevates their selenium demands, rendering them particularly susceptible to oxidative damage arising from selenium deficiency [170,173,174].

One study involved 573 Madrid schoolchildren aged 8 to 13 and found that children with excess weight (BMI > 85th percentile) had significantly lower serum selenium levels and selenium intake compared to those with normal weight. A negative correlation was observed between serum selenium and BMI, and logistic regression showed that the risk of selenium deficiency increased with BMI and decreased with selenium intake and age. Additionally, a positive correlation between serum selenium and GPx activity was found. The results suggest that overweight children, particularly those with central adiposity, have poorer selenium status, which may contribute to impaired antioxidant protection [175].

The relationship between selenium status and obesity in children is complex, involving interactions with antioxidant defenses, thyroid hormone metabolism, and inflammation. Although the precise mechanisms and directionality of these relationships require further investigation, maintaining adequate selenium levels may help mitigate some negative metabolic effects of childhood obesity. Future research should determine optimal selenium levels for supporting metabolic health in children, accounting for individual variability and the broader nutritional context. Selenium acts as both a nutrient and a potential biomarker for pediatric obesity. Its interactions with metabolic processes, dietary intake, and bioavailability are critical for understanding and managing childhood obesity. The relationship between selenium status and obesity remains unclear; further research is needed to determine whether selenium deficiency contributes to or results from obesity. These findings will inform nutritional interventions and public health strategies for preventing and managing childhood obesity, ensuring appropriate selenium intake recommendations to prevent both deficiency and excess [176,177].

#### 2.2.4. Iron

Disruptions in iron (Fe) metabolism are frequently observed in individuals, both children and adults, who are affected by overweight or obesity [178,179,180,181,182,183,184,185,186,187,188]. The WHO reports that iron deficiency (ID) is a significant concern globally, affecting 20.1% of young children in industrialized nations and 39% in developing countries. Among older children (5–14 years), ID prevalence rises to 48.1% in developing nations.

ID is a major global health concern, with higher prevalence in developing countries. A study in Thailand examined 99 children affected by overweight and obesity (ages 5–15) to assess the relationship between obesity and iron status. Results showed that 51.52% had ID, while 3% had ID anemia. Children with overweight and obesity had low serum iron and transferrin saturation but elevated serum ferritin with normal total iron-binding capacity. The study highlights that ID is more common in children diagnosed with obesity, and serum iron and transferrin saturation are better indicators of ID than serum ferritin or TIBC [189].

Iron is obtained through dietary absorption and the recycling of red blood cells by macrophages. The small intestine absorbs inorganic iron via divalent metal transporter-1 (DMT-1), which requires the conversion of ferric iron (Fe^3+^) to ferrous iron (Fe^2+^), while heme iron is transported through heme carrier protein-1. Once inside the enterocytes, heme iron is released by heme oxygenase and either stored in ferritin or transported into the bloodstream via ferroportin, an Fe^2+^-selective protein. In circulation, iron must bind to transferrin for tissue delivery, requiring oxidation by the enzyme hephaestin [189]. Beyond dietary intake, the primary source of iron is erythrocyte recycling by macrophages in the spleen, bone marrow, and liver Kupffer cells. After red blood cell breakdown, macrophages release iron into the bloodstream, where transferrin transports it to the bone marrow for new red blood cell synthesis or to storage sites like the liver [190,191].

Emerging evidence suggests a link between ID and obesity, potentially driven by obesity-induced inflammation. Adipose tissue expansion in obesity triggers chronic low-grade inflammation, characterized by an increased production of pro-inflammatory cytokines, including IL-6, IL-1, IL-8, and TNF-α, primarily by infiltrating macrophages. Adipocytes also contribute to inflammation by secreting leptin. IL-6 and TNF-α stimulate the liver’s production of acute-phase proteins such as C-reactive protein, α-1 acid glycoprotein, ferritin, and hepcidin [192]. This imbalance in adipokine production promotes persistent inflammation and metabolic disorders associated with obesity [193].

Hepcidin, a peptide hormone primarily produced by hepatocytes but also by immune and adipose cells, is a key regulator of systemic iron homeostasis [194,195,196]. Increased hepcidin expression lowers serum iron levels by binding to ferroportin, blocking iron release from macrophages, hepatocytes, enterocytes, and placental syncytiotrophoblasts [194]. Additionally, hepcidin suppresses DMT-1 expression in enterocytes, reducing dietary iron absorption. Several factors influence hepcidin synthesis, including iron status, oxygen levels, and inflammation. While iron sufficiency induces hepcidin expression to prevent excess accumulation, hypoxia inhibits it to facilitate hemoglobin synthesis. Inflammatory signals, particularly IL-6, strongly upregulate hepcidin production, leading to hypoferremia [194,195,196,197,198].

Chronic inflammation associated with obesity triggers the release of IL-6, which stimulates hepcidin production, leading to ID. This condition is prevalent in children and adolescents affected by obesity, who typically show poor responses to iron supplementation. However, these individuals tend to respond better to weight loss interventions, which help restore iron balance. Given this, it may be beneficial to incorporate serum hepcidin level measurement when evaluating children and adolescents with ID. This approach could help guide clinical decisions and improve therapeutic outcomes [199].

It seems that obesity may contribute to increased iron accumulation within tissues while simultaneously reducing circulating iron levels in the bloodstream. This imbalance can lead to excessive iron storage in tissues and a reduction in iron availability for blood cell production. The retention of iron within tissues has been linked to MetS, a condition characterized by obesity, high blood pressure, IR or type 2 diabetes, and abnormal lipid levels [200,201,202,203]. Furthermore, it is associated with the onset of nonalcoholic fatty liver disease (NAFLD), which represents the liver-related aspect of MetS.

Due to the complex interactions between obesity, iron, and inflammation, isolating the effects of each factor is challenging. Inflammatory responses can alter several iron-related indicators, particularly serum iron levels and transferrin, the protein responsible for iron transport. Some researchers suggest evaluating additional markers of iron balance, such as soluble transferrin receptor and hepcidin, the primary regulator of systemic iron homeostasis [204,205,206]. However, these indicators have limited application in clinical settings [185,188,207,208,209,210]. Meanwhile, serum ferritin, an indicator of iron reserves within tissues, is regarded as the most reliable marker for identifying ID—provided inflammation is absent or measured alongside an inflammatory protein [205,206].

Among children and adolescents with obesity, the prevalence of ID ranges from 2.0% to 4.8%, depending on the threshold used to define ferritin levels. This rate is comparable to or lower than that reported in broader studies of healthy French youth. For instance, a review by Hercberg et al. [211] found that 13.6% of French children aged 2–6 years and 3.1–15.4% of adolescent girls exhibited abnormal serum ferritin levels [211]. Similarly, Canadian adolescents with comparable lifestyles showed a 13.1% prevalence of low ferritin levels [212]. As expected, ID was more common among girls than boys.

Other research has reported an ID rate of 11.2% in adolescents diagnosed with obesity [183,186]. However, contrary to these findings, some studies suggest a higher prevalence of ID in overweight individuals. This inconsistency may be due to factors beyond body weight itself, as ID does not appear to be strictly weight-dependent. For instance, Nead et al. [179] reported an increase in ID rates from 2.1% in children with normal weight to 5.3% and 5.5% in children with overweight and obesity, respectively [179]. Additionally, gender has been identified as a significant determinant of ID risk among adolescents affected by obesity [181].

In a cross-sectional study [212] involving 502 youths affected by obesity, multivariate regression analysis demonstrated that iron storage, as indicated by serum ferritin, was associated with MetS and NAFLD risk factors. These relationships remained significant even after adjusting for weight status and the acute-phase protein fibrinogen. Future research incorporating additional iron metabolism markers may provide further insights into the mechanisms underlying these associations.

Obesity in children and adolescents can also be associated with poorer cognitive function, including lower IQ, reduced cognitive abilities, and deficits in motor and visuospatial skills. Studies show that factors like excess body fat and sedentary behavior are linked to cognitive impairments, including attention, concentration, and executive function [213]. Elevated hepcidin levels may play a significant role in this process [213].

The main sources of the discussed antioxidants are presented in Table 1.

**Table 1:** Dietary antioxidants and their content per 100 g.

Antioxidants	Food Sources	Amount/100 g
Vitamin D [94]	Fish liver oil	252 mcg
	Fish herring	23.5 mcg
	Salmon	5 mcg
	Fortified milk	2.45 mcg
	Chicken liver	2 mcg
	Butter	1.53 mcg
	Boiled egg	1.3 mcg
Vitamin C [59]	Cherry	941.1 mg
	Raw yellow pepper	201.4 mg
	Orange juice	73.3 mg
	Cashews	219.3 mg
	Papaya	82.2 mg
	Kiwi	70.8 mg
Vitamin E [88]	Wheat germ oil	149.4 mg
	Chili powder	38.1 mg
	Dried sunflower seeds	35.2 mg
	Cayenne pepper	29.8 mg
Vitamin A [62]	Cod liver oil	30,000 mcg
	Beef liver	7744 mcg
	Fortified breakfast cereals	990 mcg
	Baked sweet potatoes	961 mcg
Magnesium [147]	Whole wheat	117 mg
	Spinach	157 mg
	Quinoa	118 mg
	Almonds	76 mg
Zinc [157]	Raw oysters	91 mg
	Grilled beef	8.5 mg
	Cooked beef liver	4.5 mg
	Cooked turkey	4.5 mg
	Cooked veal	4.4 mg
	Cooked chicken liver	4.3 mg
Iron [195]	Parsley (leaves)	5.3–6.2 mg
	Peas	4.7 mg
	Potatoes	3.2 mg
	Spinach	2.8 mg

### 2.3. Other Antioxidants

Herbal medicine (HM) is a globally prevalent complementary and integrative medicine therapy for weight loss [213,214]. Its appeal stems from dissatisfaction with conventional treatments, positive past experiences, and cultural traditions [215,216]. HM’s mechanisms for weight reduction include appetite suppression, enhanced lipid metabolism, pancreatic lipase inhibition, lipolysis promotion, and inhibition of adipogenesis [217]. While some studies have examined HM use, efficacy, and safety in obese adolescents [217,218], research on the factors influencing adolescent use of HM for weight loss remains lacking. A study on 46,336 adolescents from Korea who used HM for weight loss found that factors such as household economic status, parental education, and mental health influenced HM use. Male students and those from lower economic backgrounds were less likely to use HM, while those with depression or chronic allergic diseases were more likely to. Female students affected by obesity tended to use HM more than overweight females. These findings highlight the need for targeted strategies to promote HM use and suggest that expanding health insurance coverage for weight loss interventions could be beneficial [219].

Many people are adopting healthier lifestyles by using natural ingredients rich in antioxidants to prevent and treat diseases. Antioxidants like kahweol and flavonoids such as resveratrol have shown antidiabetic, antiobesity, and antioxidant effects. The combination of resveratrol and quercetin has been found to help manage hyperglycemia and dyslipidemia in diabetic rats.

CoQ10, existing as ubiquinol or ubiquinone, is a crucial component of the mitochondrial electron transport chain, essential for cellular energy production. Statin-induced inhibition of HMG-CoA reductase is a common cause of CoQ10 deficiency, as it blocks the conversion of HMG-CoA to mevalonate, disrupting lipid metabolism. CoQ10 exhibits antioxidant effects through several mechanisms: as a cofactor and activator of mitochondrial uncoupling proteins, facilitating electron transfer; inhibiting lipid and protein peroxidation; preventing LDL oxidation; and enhancing the bioavailability of other antioxidants, including vitamins C, E, and β-carotene. Research has demonstrated that CoQ10 supplementation (100–150 mg/day) or consumption of CoQ10-rich foods can significantly improve carbohydrate metabolism, alleviate inflammation, and mitigate OS, all of which are hallmarks of obesity and MetS [220,221,222,223].

N-acetyl cysteine (NAC), a potent antioxidant, addresses the inflammatory response and oxidative damage induced by obesity. It also plays a crucial role in inhibiting lipid accumulation by targeting specific adipogenic transcription factors. Murine models have shown that NAC effectively counteracts hyperglycemia, dyslipidemia, and OS caused by a sucrose-rich diet by upregulating the expression of endogenous antioxidant enzymes [224,225,226].

Melatonin, traditionally recognized for regulating circadian rhythms and sleep, is increasingly acknowledged as a modulator of lipid metabolism, glucose homeostasis, OS, inflammation, and adipose tissue function. It influences metabolic processes by modulating melatonin receptors (MT1 and MT2), thus impacting the lipid and glucose profiles. Supplementing with melatonin (1–20 mg/day) has been shown to reduce mitochondrial damage, improve glycemic regulation, and enhance the activity of brown adipose tissue in pediatric obesity without significant adverse effects. Additionally, melatonin’s antioxidant properties have been demonstrated to reduce muscle damage following physical exertion in overweight individuals [227,228,229,230,231].

Taurine (2-aminoethanesulfonic acid), though less widely studied, has considerable effects on oxidative and metabolic stress, inflammation, insulin sensitivity, and vascular remodeling. A growing body of evidence suggests that taurine excretion is inversely correlated with BMI, blood pressure, and cholesterol levels, pointing to its potential role in obesity-related comorbidities. Supplementing taurine (3 g/day), particularly in combination with physical exercise, has shown benefits in reducing inflammation, mitigating OS, preventing endothelial dysfunction induced by high-fat diets, and improving subcutaneous adipose tissue plasticity [232,233,234,235,236,237,238,239].

## 3. Discussion and Limitations

A critical omission in many studies is the lack of discussion on potential risks associated with antioxidant supplementation. High doses of vitamin C may increase kidney stone risk in susceptible individuals [240]. Excessive intake of vitamin E has been linked to increased all-cause mortality in some populations [241]. Over-supplementation of vitamin A can lead to hepatic toxicity and teratogenic effects [242]. Excess selenium may cause neurological symptoms, while high zinc intake can disrupt copper absorption and immune function [243]. Additionally, the antioxidant paradox suggests that excess antioxidants might blunt adaptive OS responses, potentially reducing mitochondrial biogenesis and metabolic efficiency [244]. Given these potential risks, antioxidant therapy should be personalized and closely monitored.

Despite promising results, research on antioxidant supplementation in childhood obesity remains inconclusive. Several factors contribute to inconsistencies in study findings, including variability in study designs, differences in dosage, duration, and assessment methods, as well as variations in population and age groups, where younger children and adolescents show different metabolic responses to supplementation [245]. Additionally, diet and lifestyle factors play a crucial role, as the effects of antioxidants may vary depending on dietary habits, physical activity, and baseline antioxidant levels [246]. Some studies suggest significant improvements in OS markers and metabolic function, while others report no substantial benefits compared to standard interventions, highlighting the need for long-term randomized controlled trials [247]. While antioxidants hold promise in mitigating OS and improving metabolic outcomes in childhood obesity, their efficacy remains controversial. The variability in research findings, coupled with potential adverse effects, underscores the need for well-controlled clinical trials. Future research should focus on developing personalized antioxidant therapy by assessing biomarkers of OS to tailor supplementation, evaluating antioxidant supplementation alongside diet and exercise for optimal benefits, and investigating long-term safety and efficacy in diverse pediatric populations. Ultimately, while antioxidants may serve as a valuable adjunct therapy, they should be integrated within a comprehensive obesity management plan, prioritizing nutritional balance, physical activity, and clinical monitoring.

This review of existing studies on antioxidant supplementation in childhood obesity reveals a high degree of variability in findings due to differences in study design, population characteristics, and intervention protocols. While multiple studies suggest that antioxidants may help reduce OS and improve metabolic function, inconsistencies in results and methodological limitations complicate the interpretation of their overall efficacy.

Several observational studies indicate that obese children tend to have lower baseline levels of endogenous antioxidants, such as vitamins C and E, as well as trace minerals like zinc and selenium, which are essential for antioxidant enzyme activity. These deficiencies correlate with increased oxidative damage, inflammation, and metabolic dysfunction. However, interventional trials investigating the effects of antioxidant supplementation have produced mixed results. Some studies report significant reductions in OS markers, improvements in insulin sensitivity, and enhanced endothelial function, particularly with vitamin C and E supplementation. In contrast, other trials show no substantial metabolic benefits or only transient effects that do not persist after supplementation ends.

Among the antioxidants studied, vitamin C has received the most extensive investigation, with evidence suggesting its role in reducing inflammatory cytokines, improving endothelial function, and mitigating OS-related IR. However, the results for other antioxidants, such as selenium and zinc, are less conclusive, as many studies either include them in combination therapies or provide only brief analyses without clear mechanistic explanations. Selenium, for example, has been linked to improved GPx activity, yet findings on its direct impact on obesity-related OS remain inconsistent. Similarly, zinc plays a crucial role in the function of SOD, but the degree to which supplementation affects metabolic outcomes in obese children has not been systematically quantified.

Overall, while the available literature suggests potential benefits of antioxidants in childhood obesity, the heterogeneity in study designs, lack of standardized dosing protocols, and absence of large-scale meta-analyses prevent definitive conclusions. Future research should focus on well-controlled, randomized clinical trials with standardized methodologies to clarify the efficacy of individual antioxidants and their long-term effects on metabolic health.

While antioxidant supplementation has been proposed as a potential strategy to mitigate OS and metabolic dysfunction in childhood obesity, the current body of evidence remains inconclusive. Although some studies suggest that vitamins C and E, selenium, and zinc may help reduce oxidative damage, improve insulin sensitivity, and modulate inflammatory responses, conflicting findings highlight the need for caution in interpreting these results. Several trials have reported no significant metabolic benefits, while others indicate that observed effects may be transient and highly dependent on dosage, study duration, and individual metabolic responses.

Antioxidant therapy should not be viewed as a standalone intervention but rather as a potential adjunct within a broader obesity management framework. Lifestyle modifications, including dietary improvements, physical activity, and behavioral interventions, remain the most evidence-based and sustainable approaches for treating childhood obesity. Moreover, pharmacological treatments, such as metformin or GLP-1 receptor agonists, have demonstrated efficacy in managing obesity-related metabolic dysfunction and should be considered when lifestyle interventions alone are insufficient. The role of antioxidants within this context remains unclear, as few studies directly compare their effects to other treatment modalities.

Beyond efficacy concerns, several challenges must be addressed before antioxidant supplementation can be recommended in clinical practice. These include variability in bioavailability, lack of standardized dosing guidelines, and potential long-term safety concerns. High doses of certain antioxidants, such as vitamin A and selenium, have been associated with adverse effects, including toxicity and disrupted metabolic homeostasis. Additionally, individual differences in nutrient absorption and metabolism may influence treatment outcomes, raising the question of whether targeted supplementation based on OS biomarkers would be more effective than generalized recommendations.

## 4. Conclusions

In summary, the supplementation of vitamins D, C, E, and A, along with essential minerals such as magnesium, iron, selenium, and zinc, presents significant potential as a therapeutic strategy to alleviate oxidative stress, inflammation, and metabolic dysfunction in obese pediatric populations. The common imbalance between reactive oxygen species (ROS) and antioxidant defenses in childhood obesity significantly contributes to metabolic disorders, including insulin resistance and chronic inflammation. These vitamins and minerals function as potent antioxidants, neutralizing free radicals and mitigating oxidative damage to cells.

Vitamin D not only supports bone health but also enhances insulin sensitivity and reduces systemic inflammation, while vitamin E protects against lipid peroxidation and supports immune function. Vitamin C is crucial for collagen synthesis and vascular integrity, helping to reduce oxidative stress and improve endothelial function. Vitamin A, particularly in its retinoic acid form, modulates genes associated with inflammation, influencing obesity-related inflammatory markers. Minerals like magnesium, iron, selenium, and zinc are vital for metabolic health; magnesium deficiency is common in obese children and exacerbates insulin resistance, while selenium neutralizes harmful peroxides and zinc supports immune and antioxidant activities.

A key contribution of this review is its critical assessment of methodological limitations in existing studies. Unlike previous reviews that primarily discuss antioxidants’ benefits, this work highlights the need for rigorous randomized controlled trials, addresses biases in study designs, and underscores challenges related to bioavailability, dosing, and long-term safety.

While this article does not provide new experimental data and is limited by existing literature, it emphasizes the necessity for well-structured, placebo-controlled trials to explore the long-term effects and clinical efficacy of antioxidant supplementation in obese children. Moreover, comparative analyses with other obesity interventions, such as pharmacological treatments and lifestyle modifications, are essential for contextualizing their roles in broader obesity management strategies.

Ultimately, the absence of well-controlled, long-term randomized trials precludes definitive conclusions about antioxidants in childhood obesity management. Future research should prioritize large-scale, placebo-controlled studies with standardized methodologies to determine optimal dosages, long-term safety, and real-world clinical applicability.

## Data Availability

No new data were generated.
